# Privacy and Security for Resource-Constrained IoT Devices and Networks: Research Challenges and Opportunities

**DOI:** 10.3390/s19081935

**Published:** 2019-04-25

**Authors:** Shancang Li, Houbing Song, Muddesar Iqbal

**Affiliations:** 1 Department of Computer Science and Creative Technologies, University of the West of England, Bristol BS16 1QY, UK; 2 Department of Electrical, Computer, Software, and Systems Engineering, Embry-Riddle Aeronautical University, Daytona Beach, FL 32114, USA; Houbing.Song@erau.edu; 3 School of Computing, London South Bank University, London SE1 0AA, UK; m.iqbal@lsbu.ac.uk

**Keywords:** Internet of Things, privacy and security, resource-constrained devices

## Abstract

With the exponential growth of the Internet of Things (IoT) and cyber-physical systems (CPS), a wide range of IoT applications have been developed and deployed in recent years. To match the heterogeneous application requirements in IoT and CPS systems, many resource-constrained IoT devices are deployed, in which privacy and security have emerged as difficult challenges because the devices have not been designed to have effective security features.

## 1. Introduction

With the exponential growth of the Internet of Things (IoT) and cyber-physical systems (CPS), a wide range of IoT applications have been developed and deployed in recent years. To match the heterogeneous application requirements in IoT and CPS systems, many resource-constrained IoT devices are deployed, in which privacy and security have emerged as difficult challenges because the devices have not been designed to have effective security features.

Despite many security solutions being developed for the Internet, there are major concerns regarding the resource-constrained environment in IoT, including data encryption, privacy preservation, vulnerabilities, threats, attacks, and controls, among others. To address these privacy and security challenges, appropriate technologies have to be developed for resource-constrained environments in IoT.

The basic objective of this Special Issue is to report the most recent research efforts dedicated to strengthening the security and privacy solutions for resource-constrained devices in IoT and CPS systems. Specifically, each manuscript was carefully reviewed by at least two independent experts. All submissions were evaluated for their rigor and quality and their relevance to the topics proposed in this Special Issue. After a rigorous review process, 16 high-quality papers were accepted and included in this Special Issue. 

We will now briefly introduce the accepted papers.

## 2. The Papers

In the paper entitled “A Lightweight Cipher Based on SALSA20 for Resource-Constrained IoT Devices” [1], Lara et al. presented the stream cipher Generador Bits Pseudo Aleatorios (GBPA) based on the SALSA20 cipher for resource-constrained IoT devices of class 0, which provides security but reduces the requirements of the computing resources of devices in IoT.

In the paper entitled “An Incentive Mechanism in Mobile Crowdsourcing Based on Multi-Attribute Reverse Auctions” [2], Hu et al. proposed a computationally efficient online incentive mechanism that includes both a crowd worker selection algorithm and payment determination algorithm.

Bouaynaya et al. investigated formal methods to quantify and evaluate the risks associated with information systems in the paper “Exploring Risks Transferred from Cloud-Based Information Systems: A Quantitative and Longitudinal Model” [3]. Furthermore, methods for risk mitigation were also proposed.

The paper entitled “Efficient Privacy-Preserving Access Control Scheme in Electronic Health Records System” focused on the constrained device access control in e-health records (HER) systems by reducing the computational resources [4]. The proposed scheme can be utilized to provide security protection and privacy preservation in EHR systems.

Sun et al focused on a compressed-sensing-based fault-tolerant data aggregation in the paper “CS-FCDA: A Compressed Sensing-Based on Fault-Tolerant Data Aggregation in Sensor Networks” [5]. The efficient data aggregation can significantly reduce the requests for resources in IoT devices.

In the paper entitled “BeeKeeper 2.0: Confidential Blockchain-Enabled IoT System with Fully Homomorphic Computation” [6], Zhou et al. proposed a decentralized computation outsourcing scheme based on fully homographic computation. The test results demonstrated the effectiveness of the proposed scheme in resource-constrained devices.

Rahman et al. focused on the joint relay selection and power allocation for cooperative cognitive radio networks in the paper entitled “Joint Relay Selection and Power Allocation through a Genetic Algorithm for Secure Cooperative Cognitive Radio Networks” [7], which is an important part of IoT. The simulation results have shown that the proposed scheme achieved near-optimal secrecy rate performance in comparison to conventional methods.

Alromih et al. investigated a randomized watermarking technique for resource-constrained devices in the paper “A Randomized Watermarking Technique for Detecting Malicious Data Injection Attacks in Heterogeneous Wireless Sensor Networks for Internet of Things Applications” [8]. The experimental results demonstrated that the proposed scheme can provide enough security with lower energy consumption.

Fang, Yang, and Wu went through secure cost-aware data communication in IoT in the paper entitled “Security Cost Aware Data Communication in Low-Power IoT Sensors with Energy Harvesting” [9]. In this work, the authors investigated the trade-off between the size of data packets and the number of data packets.

Luo et al. focused on the low-cost security and data integrity scheme in an air quality monitoring system in the paper entitled “On the Security and Data Integrity of Low-Cost Sensor Networks for Air Quality Monitoring” [10].

Alabdulkarim et al. developed a privacy-preserving single decision tree algorithm for resource-constrained IoT devices in the paper entitled “PPSDT: A Novel Privacy-Preserving Single Decision Tree Algorithm for Clinical Decision-Support Systems Using IoT Devices” [11]. This work utilizes the homomorphic encryption cipher to protect data.

Lara-Nion et al. investigated efficient scalar multiplication for resource-constrained devices in the paper “Energy/Area-Efficient Scalar Multiplication with Binary Edwards Curves for the IoT” [12]. In this work, the proposed energy-reducing techniques can provide efficient area/energy trade-offs.

In the paper entitled “FPGA Modeling and Optimization of a SIMON Lightweight Block Cipher” [13], Abed et al. implemented an optimized lightweight SIMON cipher design for resource-constrained IoT devices. The design requires 39% less resources and 45% less power consumption.

Qin et al. proposed a lightweight anomaly detection system for IoT in the paper titled “IMLADS: Intelligent Maintenance and Lightweight Anomaly Detection System for Internet of Things” [14]. This work focused on the application level to reduce the requirements for computational resources.

Al-Otaibi et al. aimed at developing a privacy-preserving vehicular rogue node detection scheme for light devices in IoT and fog computing in the paper entitled “Privacy-Preserving Vehicular Rogue Node Detection Scheme for Fog Computing” [15]. The simulation results showed that the proposed scheme can significantly improve data processing.

Shifa et al. proposed a lightweight cipher for H.264 video in IoT in the paper entitled “Lightweight Cipher for H.264 Videos in the Internet of Multimedia Things with Encryption Space Ratio Diagnostics” [16]. This work showed the cipher’s good performance in reducing the resource consumption at the IoT application level.

## 3. Concluding Remarks

In this Special Issue, a wide range of topics are reported that cover ongoing research interests regarding security and privacy solutions for resource-constrained devices in IoT and CPS systems. The team hopes that this Special Issue will make contributions to encouraging IoT security and privacy issues.

In conclusion, we sincerely thank all researchers for their sharing of their research works to this special issue and the reviewers for volunteering their time and expertise to carefully reviewing and commenting on all submissions. We would like to thank the sensors EIC and the admin team for their continuous support and guidance. 
